# Effect of processing on the biochemical contents of *Acanthus montanus* (Nees) T. Anderson (Acanthaceae) leaves

**DOI:** 10.1002/fsn3.567

**Published:** 2017-12-11

**Authors:** Andrew Igwe, Chinedum Eleazu

**Affiliations:** ^1^ National Root Crops Research Institute Umudike Nigeria; ^2^ Federal University, Ndufu‐Alike Ikwo Nigeria

**Keywords:** drying, minerals, nutrients, polyphenols, vitamins

## Abstract

The effect of processing on the biochemical contents of *Acanthus montanus* leaves was investigated. The moisture, crude protein, lipid, fiber, ash, and total carbohydrate contents of the raw vegetable were 59.15, 1.85, 2.32, 3.76, 2.04, and 34.65 g/100 g, respectively. The saponin, alkaloid, tannin, flavonoid, phenol, and anthocyanin contents of the raw vegetable were 5.35, 4.04, 1.10, 3.53, 2.87, and 1.27 g/100 g, respectively, while it contained 2.65 mg/100 g calcium, 1.14 mg/100 g magnesium, 7.66 mg/100 g potassium, 350.75 μg/g vitamin A, 50.87 mg/100 g vitamin C, and 0.25% titratable acidity. There were significant reductions (*p* < .05) in the protein, lipid, fiber, ash, saponin, alkaloid, tannin, phenol, anthocyanin, calcium, magnesium, potassium, vitamin A, vitamin C, and titratable acidity of the boiled or boiled + sun‐dried *A. montanus* leaves; significant elevation of the moisture contents but significant reduction of the total carbohydrate contents of the boiled; and significant reduction of the moisture contents of the boiled + sun‐dried vegetable compared with the raw. There were significant increases (*p* < .05) in the total carbohydrate contents of the boiled + sun‐dried leaves; significant reductions (*p* < .05) in the moisture, saponin, alkaloid, and vitamins A and C contents of the sun‐dried vegetable; and no significant differences (*p* > .05) in the lipid, calcium, potassium, and ash, but significant increases (*p* < .05) in the protein, crude fiber, total carbohydrates, tannins, flavonoids, phenols, anthocyanin, magnesium, and titratable acidity of the sun‐dried vegetable when compared with the raw. Sun drying alone either retained or enhanced the release of some important bioactive compounds in *A. montanus* leaves. Furthermore, the reduced moisture content of the sun‐dried vegetable together with its increased titratable acidity will make the sun‐dried vegetable uninhabitable for microorganisms, thereby increasing its shelf life.

## 1 INTRODUCTION

Vegetable growing is one of the important and branches of horticulture and has been practiced in most of the countries in the world since centuries. It is one of the important income sources of mainly vegetable‐growing countries. Vegetables have been used not only for nutrition purposes, but also to meet personal and social needs such as curing diseases, beautifying the planet, and so on. They also include a lot of phytochemicals that are important for human health (Bozokalfa, Esiyok, & Asciogul, 2016; Eleazu & Eleazu, 2013a, 2013b; Hricova et al., 2016; Ipek, Turkmen, Fidan, Ipek, & Karci, 2016). In tropical Africa where the daily diet is dominated by starchy staples, African indigenous leafy vegetables are the most readily available sources of important nutrients such as vitamins, minerals, and so on. These vegetables are important commodities for poor households because their prices are more easily affordable than other food items (Eleazu & Eleazu, 2013a, 2013b; Martin & Ruberte‐Meitner, 1998). In addition, clinical trials and epidemiological studies have shown that a high dietary intake of vegetables is strongly correlated with a reduced risk of development of several chronic diseases such as cancer, type 2 diabetes, and others (Carolina et al., 2010).

*Acanthus montanus* (also called mountain thistle or alligator plant) is a perennial herb that belongs to the family of Acanthaceae. It is a striking small shrub with sparse branches and soft stem. The plant is native to Angola, Benin, Cameroon, Central African Republic, Congo, Equatorial Guinea, Gabon, Ghana, Niger, and Togo. It is also reported to be one of the threatened and underutilized species of vegetables in Africa (Ghogue, 2010; Nnamani, Oselebe, & Igboabuchi, 2015) due perhaps to its highly perishable nature.

Aside from its ornamental usage, this vegetable is also used in different homes for making soups (Djami et al., 2011) as it is considered to be nutritionally rich (Ghogue, 2010; Nnamani et al., 2015). In folk medicine, boiled forms/decoctions of this vegetable are used for different therapeutic uses such as management of diabetes, treatment of body aches and pains, cough, inflammatory/infectious diseases, cough, and others (Djami et al., 2011; Ebana et al., 2016; Ghogue, 2010; Ikezu, Ajiwe, Ilozue, & Chukwukanne, 2014; Nnamani et al., 2015). While Nigeria is a country that has abundant natural resources, it does experience occasional food crisis in terms of quantity and quality. Furthermore, there have been reported cases of malnutrition and undernutrition in Nigeria (Nnamani et al., 2015), with the food intake requirements for most Nigerians being below the international standard (Otaha, 2013).

Following the recent wind of economic recession in the developing countries, particularly in Nigeria, and its resultant effect on the purchasing power of commodities for people living in these countries, it has become obvious that the local vegetables of which the prices are easily affordable, will play beneficial roles in providing the food, nutrition, and health security of the impoverished people living in these areas (Eleazu & Eleazu, 2013b). Therefore, diversification of crops to accommodate the underutilized traditional leafy vegetables has been recommended as one of such ways of meeting the food, nutrition, and health demands of people residing in the developing countries (Nnamani et al., 2015; Okeke, Eneobong, Uzuegbunam, Ozioko, & Kuhnlein, 2008).

Despite the nutraceutical relevance of *A. montanus* leaf, there is paucity of information in literature on the residual constituents of its nutrients/bioactive compounds when cooked/boiled. This is especially of importance as cooking could induce significant changes in the chemical composition and decrease the nutritive quality and chemopreventive compounds in vegetables, so as to decrease their bioavailability when consumed.

One major challenge to ensure food security in most developing countries is making food available throughout the year. Most agricultural products are perishable, and while they are abundant in particular times of the year, they tend to be absent at other times of the year (Habou, Asere, & Alhassan, 2003), underscoring the need for a preservative method that can ensure their availability throughout the year.

One of the methods of stabilizing and extending the durability of vegetables is drying (Chan et al., 2009; Okeke et al., 2008). Drying of vegetables not only increases their shelf lives, but also ensures their availability throughout the year (Habou et al., 2003).

Given the nutraceutical relevance of this vegetable, it has become imperative to explore the preservation method that can increase its shelf life as well as retain its nutrients/bioactive compounds, for which information is lacking in literature.

Consistent with this, the present study was therefore setup to investigate the effect of boiling, drying, or their combination on the nutrient and bioactive constituents of *A. montanus* leaves.

## 2 MATERIALS AND METHODS

Fresh samples of *A. montanus* were obtained from Umuahia, Abia State, Nigeria. The nonleafy parts were removed, while the leafy parts of the vegetable were collected, properly washed, chopped into homogenous pieces, and divided into four portions. The first portion was kept as the raw (control). The remaining portions (second, third, and fourth, respectively) were divided into boiled, sun dried, and boiled + sun dried.

### 2.1 Sample treatment

For the preparation of the boiled vegetable, about 200 g of the second portion (homogenous pieces) of the vegetable was immersed in water (about 500 ml) and boiled for approximately 10 min after which they were removed and the water was drained. For the preparation of the sun‐dried vegetable, about 200 g of the third portion (homogenous pieces) of the vegetable was sun‐dried, while for preparation of the boiled + sun‐dried vegetable, about 200 g of the fourth portion (homogenous pieces) of the vegetable was immersed in water (about 500 ml) and boiled for approximately 10 min. Thereafter, they were removed from the water, water was drained, and then sun dried.

The raw and processed (boiled, sun‐dried, and boiled + sun‐dried) vegetables were then homogenized prior to analysis.

### 2.2 Proximate analysis

The method of AOAC (1984) was used for the determination of moisture, ash, protein, lipid, and crude fiber contents of the raw and processed vegetables. Total carbohydrate was obtained by difference.

### 2.3 Mineral assay

The atomic absorption spectrophotometer (Analyst 200, Perkin Elmer, Waltham, MA, USA) was used for the analysis of Mg and Ca, while the flame photometric method was used for the analysis of potassium (AOAC, 1984) in the raw and processed vegetables.

### 2.4 Phytochemical analysis

The method of AOAC (1984) was used in the determination of saponin and tannin, the gravimetric method of Harbone (1973) was used in the determination of alkaloids, flavonoids, and anthocyanin, while the method of Pearson (1976) was used in determination of the phenolic content of the raw and processed vegetables.

### 2.5 Assay of vitamins

The vitamin C content of the raw and processed vegetables was determined using the titrimetric method (Onwuka, 2005), while vitamin A was assayed using the method of Maciej and Krzysztof (2007).

### 2.6 Titratable acidity

A measured quantity (5 g) of the raw and processed vegetables was homogenized with 25 ml of distilled water. The mixture was then titrated with 0.1 M NaOH to pH 8.1 and the results were reported as % of malic acid (AOAC, 1990).

### 2.7 Statistical analysis

Data generated were analyzed statistically using the Statistical Package for Social Sciences (SPSS) version 17.0. One‐way analysis of variance (ANOVA) was used for the comparison of means. Results were considered to be significant when *p* < .05.

## 3 RESULTS

The results of the proximate composition of the raw and processed *A. montanus* leaves are presented in Table. 1. As shown in the table, the moisture, crude protein, lipid, fiber, ash, and total carbohydrate contents of the raw *A. montanus* leaves were 59.15, 1.85, 2.32, 3.76, 2.04, and 34.65 g/100 g, respectively.

**Table 1:** Proximate composition (g/100 g) of raw and processed *Acanthus montanus* leaves

Groups	MC	Protein	Lipid	CF	Ash	CHO
Raw	59.15 ± 0.17^c^	1.85 ± 0.04^b^	2.32 ± 0.06^b^	3.76 ± 0.18^c^	2.04 ± 0.06^b^	34.65 ± 1.10^b^
Boiled	69.25 ± 0.77^d^	0.79 ± 0.02^a^	1.89 ± 0.07^a^	2.72 ± 0.03^b^	1.94 ± 0.02^a^	26.13 ± 2.17^a^
Sun dried	4.5 ± 0.28^a^	1.94 ± 0.06^c^	2.32 ± 0.03^b^	3.82 ± 0.14^d^	2.02 ± 0.05^b^	89.22 ± 3.21^c^
Boiled + Sun dried	6.45 ± 0.11^b^	0.76 ± 0.04^a^	1.92 ± 0.12^a^	2.58 ± 0.07^a^	1.92 ± 0.05^a^	88.96 ± 4.44^c^

Values are means ± *SD*. Means with different superscript letters along each column are significantly different (*p *<* *.05). MC, moisture content; CHO, carbohydrates; CF, crude fiber.

Although boiling of *A. montanus* leaf significantly increased (*p* < .05) its moisture content in comparison with the raw, sun drying of the vegetable or combination of boiling and sun drying resulted in significant decrease (*p* < .05) in its moisture content compared with the raw.

The crude protein content of the boiled or boiled + sun‐dried *A. montanus* leaves significantly decreased (*p* < .05) when compared with the raw. On the contrary, the protein content of the sun‐dried leaves significantly increased (*p* < .05) when compared with the raw.

The crude lipid content of the boiled or boiled + sun‐dried *A. montanus* leaves significantly decreased (*p* < .05) when compared with the raw, while the lipid content of the sun‐dried leaves was not significantly different (*p* > .05) from that of the raw.

The crude fiber content of the boiled or boiled + sun‐dried *A. montanus* leaves significantly decreased (*p* < .05) when compared with the raw. On the other hand, the crude fiber content of the sun‐dried leaves significantly increased (*p* < .05) when compared with the raw.

The crude ash content of the boiled or boiled + sun‐dried *A. montanus* leaves significantly decreased (*p* < .05) when compared with the raw. On the other hand, the ash content of the sun‐dried leaves was not significantly different (*p* > .05) from that of the raw.

The total carbohydrate content of the boiled leaves significantly decreased (*p* < .05) when compared with the raw. On the contrary, the total carbohydrate content of the sun‐dried and boiled + sun‐dried leaves significantly increased (*p* < .05) in comparison with the raw.

The phytochemical composition of the raw and processed *A. montanus* leaves are shown in Table 2. As shown in the table, the raw vegetable contained considerable amounts of saponin. However, when the raw vegetable was processed, the saponin content of the boiled, sun‐dried, and boiled + sun‐dried *A. montanus* leaves significantly decreased (*p* < .05) when compared with the raw.

**Table 2:** Phytochemical composition (g/100 g) of raw and processed *Acanthus montanus* leaves

Groups	Saponin	Alkaloid	Tannin	Flavonoid	Phenol	Anthocyanin
Raw	5.35 ± 0.17^d^	4.04 ± 0.22^c^	1.10 ± 0.08^b^	3.53 ± 0.26^b^	2.87 ± 0.18^c^	1.27 ± 0.12^b^
Boiled	3.26 ± 0.06^b^	3.19 ± 0.10^a^	0.78 ± 0.04^a^	3.02 ± 0.12^b^	2.11 ± 0.05^b^	0.88 ± 0.08^a^
Sun dried	5.01 ± 0.28^c^	3.54 ± 0.06^b^	1.56 ± 0.03^c^	4.27 ± 0.14^c^	3.42 ± 0.05^d^	1.98 ± 0.35^c^
Boiled +Sun dried	3.22 ± 0.11^a^	3.16 ± 0.10^a^	0.76 ± 0.14^a^	2.97 ± 0.09^a^	2.06 ± 0.07^a^	0.88 ± 0.04^a^

Values are means ± *SD*. Means with different superscript letters along each column are significantly different (*p *<* *.05).

As shown in Table 2, the raw *A. montanus* leaves contained considerable amount of alkaloids. However, boiling, sun drying, or combination of boiling and sun drying of the vegetable resulted in significantly decreased alkaloid content (*p* < .05) compared with the raw.

Results shown in Table 2 indicated that boiling or combination of boiling and sun drying resulted in significant decreases (*p* < .05) in the tannin content of the *A. montanus* leaves compared with the raw. In contrast, sun drying alone significantly increased (*p* < .05) the tannin content of the vegetable when compared with the raw.

As shown in Table 2, the raw vegetable contained considerable amount of flavonoids. Boiling alone did not significantly affect (*p* > .05) the flavonoid content of *A. montanus* leaves. On the other hand, sun drying alone significantly increased (*p* < .05) the flavonoid content of the vegetable compared with the raw, whereas combination of boiling and sun drying significantly (*p* < .05) decreased the flavonoid content of the vegetable compared with the raw.

As shown in Table 2, boiling of the vegetable or combination of boiling and sun drying significantly decreased (*p* < .05) the phenol content of *A. montanus* leaves compared with the raw. On the contrary, sun drying alone significantly increased (*p* < .05) the phenol content of the vegetable compared with the raw.

Results presented in Table 2 indicated that boiling or combination of boiling and sun drying resulted in significant decreases (*p* < .05) in the anthocyanin content of *A. montanus* leaves compared with the raw, whereas sun drying alone significantly increased (*p* < .05) the anthocyanin content of the vegetable compared with the raw.

The mineral composition of the raw and processed *A. montanus* leaves are shown in Table 3. As shown in the table, whereas boiling or combination of boiling and sun drying resulted in significant (*p* < .05) decreases in the Ca, Mg, and K contents of *A. montanus* leaves compared with the raw, sun drying alone resulted in significant increases (*p* < .05) in the Mg content of the vegetable, while it did not significantly affect (*p* > .05) the Ca and K contents of the vegetable compared with the raw.

**Table 3:** Mineral composition (mg/100 g) of raw and processed *Acanthus montanus* leaves

Groups	Calcium	Magnesium	Potassium
Raw	2.65 ± 0.18^b^	1.14 ± 0.14^b^	7.66 ± 0.00^c^
Boiled	2.37 ± 0.12^a^	0.94 ± 0.07^a^	6.30 ± 0.26^b^
Sun dried	2.62 ± 0.14^b^	1.19 ± 0.17^c^	7.62 ± 0.05^c^
Boiled + Sun dried	2.38 ± 0.06^a^	0.94 ± 0.04^a^	6.20 ± 0.48^a^

Values are means ± *SD*. Means with different superscript letters along each column are significantly different (*p *<* *.05).

The vitamin composition of the raw and processed forms of *A. montanus* leaves are shown in Table 4. As shown in the table, the raw vegetable contained 350.75 ± 12.34 μg/100 g vitamin A and 50.87 ± 9.26 mg/100 g vitamin C, the boiled vegetable contained 40.92 ± 8.12 μg/100 g vitamin A and 6.03 ± 0.66 mg/100 g vitamin C, the sun‐dried vegetable had 10.59 ± 3.36 μg/100 g vitamin A and 13.39 ± 1.22 mg/100 g vitamin C, and the combination of boiled and sun‐dried vegetable had 10.52 ± 1.77 μg/100 g vitamin A and 6.01 ± 0.13 mg/100 g vitamin C, respectively.

**Table 4:** Vitamin composition of raw and processed *Acanthus montanus* leaves

Groups	Vitamin A (μg/g)	Vitamin C (mg/100 g)
Raw	350.75 ± 12.34^d^	50.87 ± 9.26^c^
Boiled	40.92 ± 8.12^c^	6.03 ± 0.66^a^
Sun dried	10.59 ± 3.36^b^	13.39 ± 1.22^b^
Boiled + Sun dried	10.52 ± 1.77^a^	6.01 ± 0.13^a^

Values are means ± *SD*. Means with different superscript letters along each column are significantly different (*p *<* *.05).

**Figure 1:**
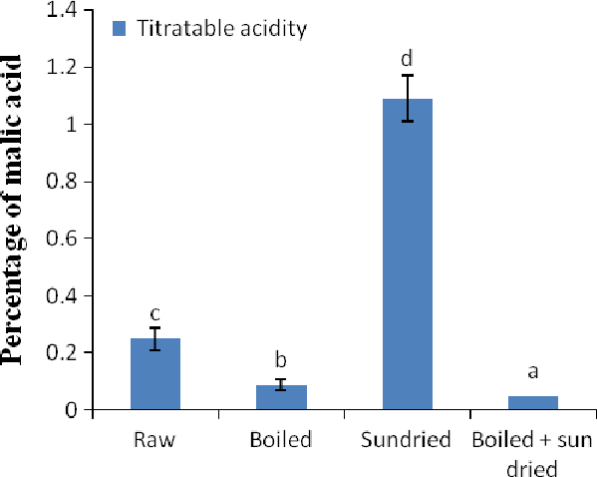
Titratable acidity in raw and processed *Acanthus montanus* leaves. Values are means ± *SD*. Means with different superscript letters (^a‐d^) are significantly different (*p* < .05)

The titratable acidity (TA) in the raw and processed vegetables is shown in Figure 1. As shown in the figure, the raw vegetable contained 0.25 ± 0.04% TA, the boiled vegetable contained 0.09 ± 0.02% TA, the sun‐dried vegetable contained 1.09 ± 0.08% TA, and the combination of boiled and sun‐dried vegetable contained 0.05 ± 0.00% TA. There were significant decreases (*p* < .05) in the TA of the boiled and boiled + sun‐dried vegetable compared with the raw, but significant increases (*p* < .05) in the TA of the sun‐dried vegetable compared with the raw.

## 4 DISCUSSION

Values obtained for most of the parameters on the proximate composition of *A. montanus* leaves were higher than previous reports of Nnamani et al. (2015) on the proximate composition of this plant except moisture in which the values obtained were lower than that reported by Nnamani and colleagues.

The decreased moisture content of the sun‐dried and boiled + sun‐dried vegetable could be attributed to loss of water during the drying process.

The decreased protein content of the boiled or boiled + sun‐dried vegetable suggests denaturation of some of the cellular proteins during the process of boiling of the vegetable (Khairul, Khan, Sarkar, Nurul, & Sarkar, 2013). This statement is further buttressed by the increased protein content of this vegetable when it was subjected to only sun drying, and in which increase could have arisen from increased release of nitrogen during the digestion process. The current study shows that sun drying of *A. montanus* leaf enhances its protein content.

Generally vegetables are known to contain small quantities of lipids. The decrease in the lipid content of the boiled or boiled + sun‐dried *A. montanus* leaves could be attributed to some lipids that may have melted into the boiling water. On the other hand, the nonsignificant difference in the lipid content of the sun‐dried vegetable when compared with the raw could be attributed to the low temperature of sun drying that may have left the ester linkages holding the lipid molecules in the vegetable intact.

Crude fiber represents the portion of food not used up by the body but mainly made up of cellulose with a little lignin (Lilian, 2002). Diets rich in crude fiber have also been shown to be beneficial as they delay the digestion of starch to simple sugars through inhibition of pancreatic amylase, which is an important factor in the management of diabetes (Eleazu & Eleazu, 2012). The decrease in the crude fiber content of the boiled or boiled + sun‐dried *A. montanus* leaves could be attributed to some soluble carbohydrates present in the vegetable that may have leached into the boiling water. However, the increased crude fiber content of the sun‐dried vegetable suggests increased release of some insoluble carbohydrates from their food matrix. The current study reveals that sun drying of *A. montanus* leaf increases its crude fiber content.

The decrease in the ash (total mineral) content of the boiled or boiled + sun‐dried vegetable could be attributed to the leakage of some minerals into the boiling water. On the other hand, the nonsignificant difference in the ash content of the sun‐dried vegetable compared with the raw suggests that sun drying of this vegetable retains its mineral content.

The increased total carbohydrate content of the sun‐dried and boiled + sun‐dried vegetable is simply due to their loss of moisture during the drying process, whereas the decreased total carbohydrate content of the boiled vegetable is due to increased moisture content during the boiling process.

Saponin possesses the potentials of precipitating and coagulating red blood cells. Other characteristics associated with saponin include formation of foams in aqueous solutions and cholesterol‐binding properties and bitterness (Awa & Eleazu, 2015). The considerable amount of saponin in the raw vegetable suggests that it might possess these properties. The decreased saponin content of the boiled, sun‐dried, and boiled + sun‐dried *A. montanus* leaves indicates that the processing methods used in this study decreased the saponin content of *A. montanus* leaves.

Alkaloids are the most efficient therapeutically significant plant substances. Pure isolated alkaloids and their synthetic derivatives are used as basic medicinal agents because of their analgesic, antispasmodic, and antimicrobial properties (Awa & Eleazu, 2015). The considerable amount of alkaloids in the raw *A. montanus* leaves suggests that the raw vegetable could possess any or all of these therapeutic properties. Although boiling, sun drying, or their combination decreased the alkaloid content of this vegetable, the amounts obtained which could be considered to be high could be one explanation for the medicinal/therapeutic usage of the boiled/decoctions of this vegetable in folklore medicine.

Tannins are water‐soluble high‐molecular‐weight phenolic compounds found in many plants that are important in herbal medicine due to their wound healing properties (Eleazu, Eleazu, Awa, & Chukwuma, 2012) and antioxidant properties.

Flavonoids and phenolic acids are known to possess antioxidant activities due to the presence of hydroxyl groups in their structures and their redox properties (Eleazu, 2016; Ukwe & Ubaka, 2011). In addition, flavonoids as the largest group of phenolics found in fruits, vegetables, and other plant parts have been linked to reducing the risk of major degenerative diseases (Eleazu, Eleazu, et al., 2012).

These polyphenols (tannins, flavonoids, and phenols) have also been associated with hypoglycemic activity due to their ability to inhibit brush border enzymes (α‐amylase, α‐glucosidase, and others) (Eleazu, 2016; Eleazu, Eleazu, et al., 2012; Ogundajo, Kazeem, Owoyele, Ogunmoye, & Ogunwande, 2016; Ukwe & Ubaka, 2011). Therefore, high amounts of these polyphenols in the raw vegetable could have contributed to the hypoglycemic, α‐amylase, and α‐glucosidase inhibitory properties of this vegetable as previously reported (Ogundajo et al., 2016; Ukwe & Ubaka, 2011).

It is noteworthy that one of the ethnomedicinal uses of the boiled form/decoctions of this vegetable is in the management of diabetes mellitus due to its ability to inhibit these brush border enzymes as supported by experimental studies (Ogundajo et al., 2016). Although boiling decreased the amounts of these polyphenols in this vegetable, the amounts obtained for most of these polyphenols in the boiled vegetable which could be considered to be high could have contributed to the hypoglycemic action of the boiled form/decoctions of this vegetable as used in folklore (Ogundajo et al., 2016; Ukwe & Ubaka, 2011). In addition, the findings of this study revealed that sun drying of this vegetable resulted in increased levels of these polyphenols in *A. montanus* leaf, a finding that may be of benefit to people with type 2 diabetes mellitus.

Anthocyanins belong to the class of flavonoids commonly found in plants that confer them with color and antioxidant activities (Karau, Njagi, Machocho, Wangai, & Kamau, 2012; Mary, 2004; Zahid et al., 2015). Since the medicinal properties of this vegetable have been associated with the alkaloids and the phenolic phytochemicals that are present in it, the findings of this study revealed that sun drying of this vegetable will help to harness its medicinal potentials.

Calcium, magnesium, and potassium are among the seven essential macrominerals. Calcium is needed for blood clotting and functioning of some enzymes. Ca also plays significant roles in bone formation and neuromuscular function (Eleazu, Amadi, & Iwo, 2012). The recommended daily allowance (RDA) of Ca is 0.5 g/day for adults and 1 g/day for children (Vasudevan, Sreekumari, & Vaidyanathan, 2011).

Magnesium is important in muscle relaxation. The RDA of magnesium is 400 mg/day and a lower than normal dietary intake of Mg can be a strong risk factor for hypertension, cardiac arrhythmias, ischemic heart disease, atherogenesis, and sudden cardiac death (Eleazu, Amadi, et al., 2012).

Potassium is a mineral and an electrolyte that is essential for cardiac and tissue health, skeletal contraction, and gastrointestinal function. The RDA of potassium is 3–4 g/day (Vasudevan et al., 2011). Optimum potassium levels tend to reduce the adverse effect of sodium on blood pressure and also decreases the risk of cardiovascular disease, osteoporosis, and kidney stones (Vasudevan et al., 2011).

The findings of this study revealed that sun drying of this vegetable leads to increased release of its Mg, while it retains its Ca and K contents. This finding therefore explains the nonsignificant effect in the ash content of this vegetable when it was sun‐dried. However, with reference to the RDA of calcium, magnesium, and potassium in adults, the findings of this study showed that consumption of 100 g of the processed *A. montanus* may not meet with this requirement and would thus require dietary supplementation from other sources.

Vitamin A is a collective name for a group of lipophilic biomolecules that are required by humans to perform different vital metabolic functions (Ramadhan & Ian, 2012). The RDA of this vitamin is 400–600 μg/day for children and 750–1,000 μg/day for adults.

Although vitamin A is known to be heat stable, it has been established that temperatures above 40°C tend to slowly degrade and break down retinol which explains the loss of this vitamin in the cooked vegetable compared with the raw. Furthermore, the loss of this vitamin in the sun‐dried vegetable compared with the raw and the higher loss obtained in the sun‐dried vegetable when compared with the boiled vegetable could be attributed to the effect of oxidation (during exposure to air) and light, vitamin A being photolytic. Any one or a combination of these factors could have contributed to the decreased levels of this vitamin following boiling, sun drying, or combination of boiling and sun drying of *A. montanus* leaves compared with the raw. However, going by the RDA of vitamin A in children (400–600 μg/day) and adults (750–1,000 μg/day), the findings of this study showed that consumption of 100 g of the boiled, sun‐dried, or boiled + sun‐dried vegetable would meet with these requirements, and this is an important finding in this study.

Vitamin C functions as one of the principal antioxidant vitamins that protect the human tissue against free radical damage. It does this by giving up electrons to provide stability to reactive species such as reactive oxygen species. The decreased levels of vitamin C following boiling, sun drying, or their combination in the *A. montanus* leaves could be attributed to the heat labile nature of vitamin C. In addition, the higher loss of this vitamin following boiling or its combination with sun drying of *A. montanus* leaves when compared with the sun‐dried leaves only could be attributed to the water solubility of this vitamin which may have resulted in its leakage into the boiling vessel. Going by the RDA of vitamin C (75 mg/day), the findings of this study showed that consumption of the processed *A. montanus* leaves will not meet with this requirement but would require dietary supplementation from other sources or eating of the fresh leaves.

Titratable acidity is a quantification of the hydrogen ions that are consumed by titration with a standard base to an end point. It gives an idea of the amount of acid that is present in a solution. The decreased TA in the boiled and boiled + sun‐dried vegetable when compared with the raw suggests that boiling of the vegetable or its combination with sun drying decreased the acidity of the vegetable. On the contrary, the increased TA in the sun‐dried vegetable suggests increasing dry matter contents of the samples or release of some organic acids during the process of sun drying that may have increased the acidity of the vegetable. While the increased TA in the sun‐dried vegetable could cause unpleasant taste in the vegetable, this increase is desirable as together with its low moisture content it will make the sun‐dried vegetable uninhabitable for microorganisms, thereby increasing its shelf life.

To the best of our knowledge, this is the first holistic documentation on the proximate, phytochemical, minerals, vitamins, and TA contents of *A. montanus* leaves and the effect of processing on these components of this vegetable.

## 5 CONCLUSION

The study revealed that sun drying of *A. montanus* leaves retained the calcium, potassium, and lipid contents of this vegetable, while it increased its protein, total carbohydrates, crude fiber, tannin, flavonoid, phenol, anthocyanin, magnesium, and TA contents. Finally, the reduced moisture content of the sun‐dried vegetable together with the increased TA will make the sun‐dried vegetable uninhabitable for microorganisms, thereby increasing its shelf life.

## CONFLICTS OF INTEREST

None declared.
